# Qualitative data regarding the experiences of pregnant women with lupus in Brazil

**DOI:** 10.1016/j.dib.2020.106606

**Published:** 2020-12-02

**Authors:** Larissa Rodrigues, Vera Lucia Pereira Alves, Maria Margarida Fialho Sim-Sim, Fernanda Garanhani Surita

**Affiliations:** a University of Campinas, Brazil; b University of Évora, Portugal

**Keywords:** Pregnancy, Systemic lupus erythematosus, Woman, Mental health, Perinatal, Qualitative research

## Abstract

A qualitative design was performed as individual face-to-face interviews with each participant, following a semi-structured script based on open questions. Participants were interviewed at a specialized clinic, where, during prenatal care, women with stable systemic lupus erythematosus disease were received scheduled consultations. The sample was intentionally composed of women who attended a specialized high-risk clinic, from July 2017 to July 2018. Participants (*N* = 26) were interviewed in-depth, without refusal. A thematic analysis, according to the 7 steps of the qualitative analysis, was performed. Before conducting interviews, the researcher went through a period of environmental adaptation to the clinic, following a service observation script and maturing the open consultation script questions, to deepen the themes derived from these women's perceptions during the individual interview. Two authors analyzed the material, which was recorded as audio and transcribed in full; later, the material that was organized in the NVIVO 11 software was validated.

## Specifications Table

**Table:** 

Subject	Obstetrics, Gynecology and Women's Health
Specific subject area	Women's health in high-risk, outpatient prenatal care
Type of data	Tables and Figures Interview script Observation script Maps
How data were acquired	The interviews were performed face-to-face, among 26 pregnant women with lupus. Interviews were recorded by Super Voice recorder and transcribed into text manually. (App Super Voice recorder available at https://play.google.com/store/apps/details?id=com.meihillman.audiorecorder&hl=en_US&pli=1)
Data format	Raw data. The complete raw data are only for peer-review and in Portuguese. However, a summary of the participants' statements divided into the questions used in the interview script is made publicly available (Table 3) The analyzed data gave rise to categories of results and are presented in an article published in the Journal of Midwifery (Elsevier) DOI: https://doi.org/10.1016/j.midw.2020.102715.
Parameters for data collection	The interviews occurred on the same day that the patients received prenatal consultations and were conducted in a private room, which was prepared to receive each participant after acceptance. This room contained two chairs facing each other (interviewee and interviewer), without tables, clipboards, or any material that kept the two people away or indicated power.
Description of data collection	The first author (L. R.) collected data by performing 1 interview with each participant, using a flexible guide and 6 open-ended questions. Before each interview, a relationship was established, creating an atmosphere of empathy, trust, and responsiveness. Participants received an explanation of the research topic, a description of the study objectives, and the rights of the parties involved, with sociodemographic data being collected from then on. Written permission was requested to use a recorder. Behavioral and intervening aspects were recorded in a field diary.
Data source location	The University of Campinas- Center for Integral Attention to Women's Health (CAISM)Campinas/State of Sao Paulo/Country: Brazil. Latitude: −22.9064, Longitude: −47.0616 22° 54′ 23″ south 47° 3′ 42″ west
Data accessibility	With the article
Related research article	Rodrigues L, Alves VLP, Sim-Simc MMF, Surita FG. Perceptions of women with systemic lupus erythematosus undergoing high-risk prenatal care: A qualitative study. Midwifery. 2020 May 13;87:102,715. https://doi.org/10.1016/j.midw.2020.102715. Epub ahead of print.

## Value of the Data

• The perspectives held by women with lupus, regarding pregnancy, is little explored [1], [2], [3].
• Health professionals can benefit from this data, by developing roadmaps for issues that can enhance the perspective of the women they care.
• Health professionals can use an observational script model in research or assistance fields.
• The replication of this method, using other populations, with different or similar characteristics in other settings, can facilitate the understanding of their experiences.
• Knowing these data can lead the health professional to reflect on the non-adherence to medication or contraceptive treatments, by women with lupus.
• The data provided knowledge regarding the characteristics associated with the participants at the research location, and health professionals can identify similarities among women treated by other services, to perform a naturalistic generalization of the research results.

## 1 Data Description

Table 1 - Characteristics of the participants [3].

A description of the study participants' sociodemographic characteristics can be viewed in Table 1 from the article “Perceptions of women with systemic lupus erythematosus undergoing high-risk prenatal care: a qualitative study”, available at https://www.sciencedirect.com/science/article/abs/pii/S0266613820300887?dgcid=rss_sd_all.

Table 2 - Raw data for various participant characteristics.

**Table 2:** Raw data for various participant characteristics.

Interview	Occupation/work	Gestational age (weeks + days)	Disease manifestation
1	cashier	29	pseudotumor in the eyelid joint pain, swelling
2	caregiver for the elderly	33 + 3	skin rash
3	manager	36 + 6	skin rash
4	call-center operator	32 + 4	idiopathic thrombocytopenic purpura renal insufficiency
5	unemployed	30	uveitis
6	saleswoman in a furniture store	32 + 5	idiopathic thrombocytopenia purpura skin rash
7	housewife	26 + 6	ulcer on the palate recurrent fever face rash
8	salesperson in a clothing store	30	skin rash (story of 2 young sisters who suffered strokes) anti-nuclear and anti-Ro factor antibody positive
9	hospital receptionist	31	glomerulonephritis (required peritoneal dialysis) and osteopenia
10	worked with handicrafts, now unemployed	30 + 4	early childhood pain crises
11	waitress	28 + 3	joint pain
12	cold-storage assistant	30 + 6	deep vein thrombosis
13	nurse	34	depression
14	retired due to disease	30	stroke
15	supervisor of children	29 + 4	thrombocytopenia, diffuse bleeding
16	industrial production-line worker	24 + 6	erythema on the skin (whole body) focal rash on the face
17	retired due to disease	26	nephritis joint pain
18	manicurist	30	nephritis and thrombocytopenia, deep vein thrombosis
19	dental assistant	29 + 1	head and face lesions
20	accounting clerk	30	anti-phospholipid antibody syndrome brain stroke
21	clerk	28 + 6	skin rash, joint pain
22	nutritionist	35	joint pain swelling
23	cleaning lady	27 + 3	recurrent fever skin rash
24	unemployed engineer	28	skin rash
25	housewife	27 + 5	recurring pain meningitis fever
26	self-employed (sales)	27	pain in arms, swelling, Raynaud's phenomenon, and systemic arterial hypertension

This table shows some raw data about the characteristics of the participants.

Table 3 – Raw data of the speeches of the participants' responses.

**Table 3:** Raw data from the participants’ responses.

Interviewed	Speech
	1) Tell me how you feel about having an autoimmune disease and 2) Tell me about the experience of being pregnant with this disease:
**I18** 55 minutes 21 seconds	She needed to be hospitalized, and the doctor said, “So, do you accept?” I said, “There is no way not to accept it, doctor.” Imagine me not accepting and having a thrombosis inside my house without being treated, and I lose my son. How will my conscience look afterwards? Just because I was proud to not want to be hospitalized?
**I2** 11 minutes 51 seconds	And after a while, I found out that I was pregnant. At first, I was scared because my fiancé, who really wanted to be a father…because I will be very sincere, I was not preventing myself [from pregnancy] because the doctor said it would not be good for me to take contraceptives…he told me it was better to avoid…and this contraceptive could make the disease worse.
**I13** 37 minutes 15 seconds	In the beginning, it was an impact because the hematologist always told me not to get pregnant because lupus affects, attacks the fetus, and aborts. And I cannot take any type of contraceptive or hormone because I am predisposed to having APS. Because I have that positive antibody…Then, he said that if I happened to ovulate, get pregnant, and everything, I wouldn't even find out about the pregnancy because I was going to abort before…And then I got pregnant. So, it was a surprise. It was a surprise. It was not planned at all. But God knows what he does.
**I16** 35 minutes 06 seconds	Look, you have one more complication. So, I think you're scared. Having it again, there will be complications. At first, it was difficult for me to accept. Wow, it took me about two months to accept the pregnancy. But today I am very happy. Now that I'm going to have a child.
**I23** 17 minutes 25 seconds	It wasn't planned; it was a surprise. I did not use any contraceptives because, in my other relationship, I had relationships normally, did not avoid, and did not gain a child. And then, I believed that I was not coming. For me, the drugs I take for lupus had wiped out my uterus. I thought I was not pregnant anymore. That's what I put in my head. And I trusted that. Then, in the new relationship, came the pregnancy.
**I25** 14 minutes 39 seconds	I didn't understand much about gravity. But I'm taking the pregnancy peacefully.
**I26** 40 minutes 08 seconds	The doctor who attended me when I checked in to do the curettage [for the previous abortion] said that I was crazy about getting pregnant because I have lupus. I couldn't put my life at risk…He spoke to the other doctors, “Ah, this one, she lost [referring to the abortion], but she wants to get pregnant.” You know? That I was aware of the problem I had, but I still wanted to try. He wanted to rule my life. I was there suffering; because it is a loss, a pregnancy that you want, and the person treats you that way. I even complained about him here.
	3) Tell me about your follow-up during pregnancy:
**I5** 22 minutes 34 seconds	Because I thought I was going to get there [at the clinic for consultation] and I wasn't going to listen to the heart, I was already prepared to hear that it hadn't worked out.
**I6** 38 minutes 27 seconds	My concern is this: being born perfect. The drugs I take harm him. Because I think this is the concern of people who are mothers: doing harm to the baby, reaching something. So, sometimes, listening to what women say [about the healthy children of lupus mothers] more relieved and the doctor said that lupus sleeps, but there are times when he wakes up, understand? So, each case is different.
**I6** 38 minutes 27 seconds	For me, I have only the pregnancy, and that's it. Because if we don't go crazy, because if you go online and search, “can women with lupus get pregnant?” you see everything. So I prefer not to see it. If it's a phase, let's go, it will pass, and that's it…the only major concern is with the baby…if it will be perfect, if…that's why all the ultrasounds that I do, I ask the doctors, “Is everything ok?” You can talk, right, because…pregnancy…there are people who are healthy, the child comes with a problem.
**I8** 22 minutes 42 seconds	So, I don't know why. Sometimes I think it's a lot [care in the health service] because with the results of these exams today, I may not have anything, but tomorrow I may have. But at the same time, I think that is too much. I know it is necessary, you know? So, I don't know…[PAUSE].
**I10** 31 minutes	He's chubby. He'll be born big. I was a little afraid because it took a long time to do an ultrasound. I asked if I could see the brain. They said that I couldn't see it anymore [because I was at an advanced gestational age]. Then I just cry out to God and ask him to come with good health. Counting on the grace of God.
**I13** 37 minutes 15 seconds	And with pregnancy came even more health. Thank God. Because when I got pregnant…Some of my tests were changed. After I got pregnant, all of my exams became normal. And the FAN was inactive after the pregnancy. So, I think that for me, it was God who sent this son because my health has improved a lot, my heart.
**I14** 49 minutes 52 seconds	Now, I don't even feel like I have lupus. It looks like I don't have lupus [in pregnancy] Because everything the doctor said I couldn't do, I do it. Do not speak [to the doctors]. He was talking to me about the sun, which was meant to be protective. I don't pass anything. He said it might come out. There was no stain. I don't feel any pain.
**I24** 16 minutes 20 seconds	I don't think I need to [undergo all of the treatments received during pregnancy].
	4) Tell me about your relationships since you discovered the disease and pregnancy:
**I14** 49 minutes 52 seconds	My mother treats me like a patient. She is always worried about me. My husband doesn't anymore. He treats me like a normal person. To walk, he wants to pull me because he wants me to walk fast. When we are like this [relaxed], he comes and jumps on my lap, and sometimes my leg hurts. My mom gets angry but doesn't speak to him. Then she gets mad and comes to talk to me. And then I talk to him. But because he treats me like that, I feel normal, and it's better than feeling sick.
**I25** 14 minutes 39 seconds	This pregnancy was to strengthen our union, only now I decided that it is the last one. That I'm not taking any more chances because I know it's dangerous.
**I26** 40 minutes 08 seconds	My husband, at the beginning, did not accept [the pregnancy]. He was unsure. I wanted to tell him [about the pregnancy] things, and he would say, “I don't know what you want to tell it for. You don't even know if you're going to carry this pregnancy forward.” So he was looking forward to the abortions, which had already happened twice. He didn't want to get attached. But at first, it was difficult. I really felt his contempt in that part. But I understand, it is for fear of having that suffering again.
	5) Tell me about your life today:
**I7** 49 minutes 59 seconds	There's that wall, like, I can't go from there. But you can get there, right? So, like a normal person, right? But you don't have to keep saying, “Ah, but you can't.” Like, instead of helping you, that you're capable, [some people] keep putting you down.
**I12** 28 minutes 39 seconds	I hate taking phone calls at night. The ringing of the phone. That feeling comes. There comes that horrible feeling of receiving news of death.
**I25** 14 minutes 39 seconds	The disease is visible. Because I was skinny, and suddenly I was swollen, very fat. They played games at school too. They asked if I was pregnant at the time. It upset me a lot. But then I started taking it normal. Nowadays, I already have another thought. I think if you want to be with me, you will live my way, not me in her way.
	6) Is there something else you want to talk about?
**I6** 38 minutes 27 seconds	When I was about 19 years old, the doctor said that to have a child, I would have to have a treatment, so much that I never took contraceptives. I never took them. So, when I found out I was pregnant, I said, “my God is a miracle,” because I never took it, never used a condom, and never got pregnant. So, for me, it was a miracle, a very good feeling…at first I didn't believe it. I only believed it when I saw the ultrasound, that I saw that little thing there.
	
**I25** 14 minutes 39 seconds	People asked how I was, and I said, “I'm fine, I have nothing.” But it is difficult because lupus is not very popularly known. People did not know what lupus was. There, they were afraid. I already lost a lot of friendship because they thought it was contagious. Because it was a total change, mainly of the body.
**I25** 14 minutes 39 seconds	I once stopped the medications. When I lost my mom. I was disgusted. But then, I realized later that it's no use. My luck was that lupus did not activate. But then I came back. So, I just abandoned the medication, but the doctor did not.
**I2** 11 minutes 51 seconds	Work takes away my peace. With the pregnancy, the relationship with the company worsened even more.
**I7** 49 minutes 59 seconds	No, I don't work now. I quit because I started to feel really bad, and as I was working in a refrigerator. When I started the treatment of lupus, I worked in a very closed place and got very sick; got everything. It was flu, pain, you know? Fever, almost every day, and then I walked away. Then, the doctor discharged me. I filed a lawsuit because there was no way I could go back there because the place was closed, and they didn't want to move me to a more open place, you know? But then I went to work in the refrigerator. It got worse.
**I23** 17 minutes 25 seconds	With work, it is horrible because, in a farm, I am homely. My friends are doing my job because I can't do it. I can't do it. We need the money to survive because my husband is unemployed.

This table shows a summary of the participants' responses divided by the questions in the script used, previously the construction of categories in the data analysis process.

A figure demonstrating the interview reports and the category construction process can be found as Fig. 1, in the article “Perceptions of women with systemic lupus erythematosus undergoing high-risk prenatal care: a qualitative study” [3], available at https://www.sciencedirect.com/science/article/abs/pii/S0266613820300887?dgcid=rss_sd_all.

**Fig. 2:**
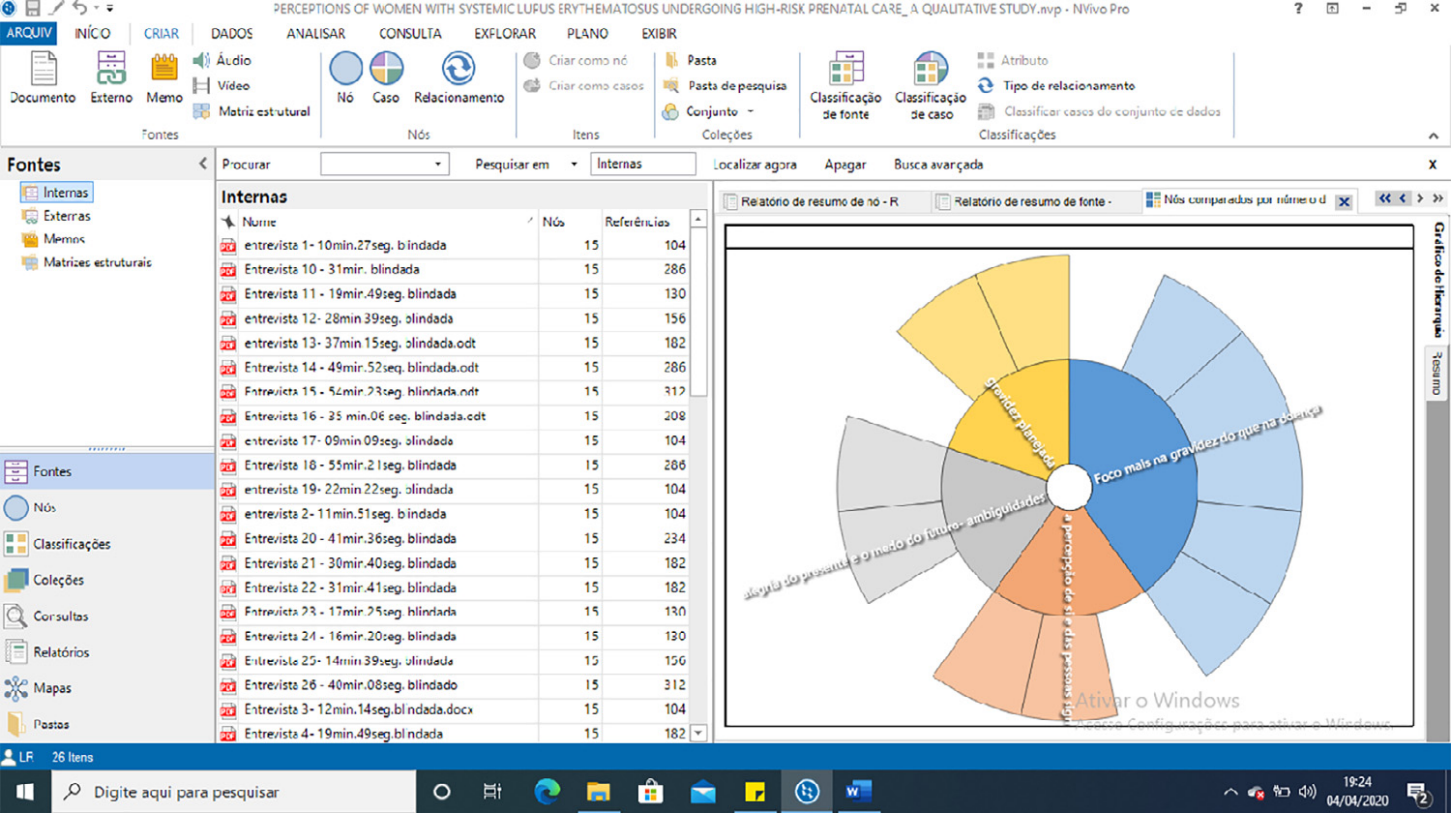
Image of the generation of codes and graphics for the NVIVO 11 categories.

Fig. 2 represents a photograph of the data processed using the NVIVO11 software, after being manually analyzed by two researchers, as units of meaning that will be used as nodes. The transcribed and recorded materials of the interviews were coded by the software, showing that the references to speech vignettes chosen for each category were included and coded by the software. NVIVO identified references from all interviews, for all generated nodes and subnodes, as shown in Fig. 2.

**Fig. 3:**
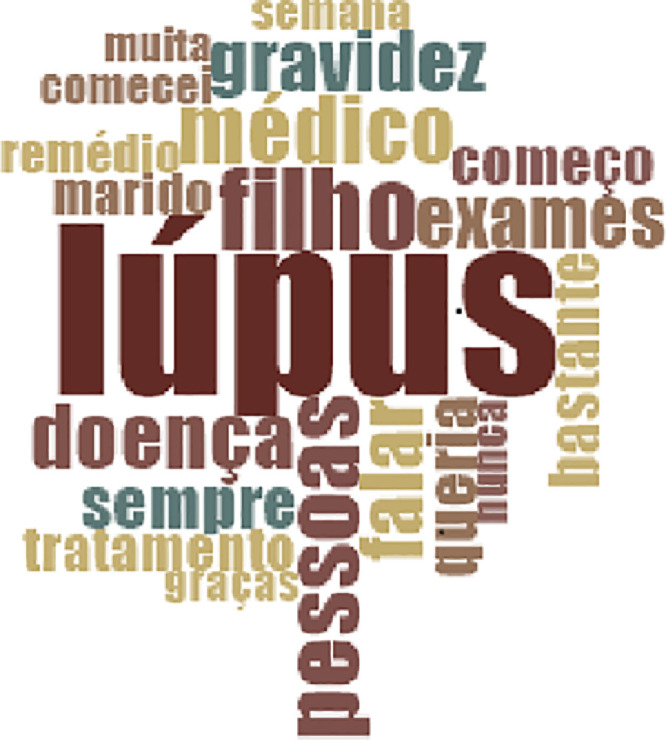
Word cloud formulated through NVIVO software.

This figure shows that the bigger the word appears in the figure, the greater its frequency of appearance in the participants' speeches.

Supplementary material.

Appendix 1- The service coverage map

A geographical and political perspective of the survey location, including a map of the country, a map of the state, and a map of the regional metropolis.

Appendix 2- Guide for the observation of setting during environmental adaptation

The guide used to observe the activities during field research.

Appendix 3 - Semi-structured script, with six open questions

The guide used to conduct interviews.

## 2 Experimental Design, Materials and Methods

A detailed description of the experimental design, materials, and methods can be found in the article “Perceptions of women with systemic lupus erythematosus undergoing high-risk prenatal care: a qualitative study” [3], available at https://www.sciencedirect.com/science/article/abs/pii/S0266613820300887?dgcid=rss_sd_all.

### 2.1 Study design

The primary study was designed using the qualitative method, specifically for a clinical setting, where the practice of patient care is routine [4, 5]. The foundation of the method includes three pillars that represent three attitudes of the researcher. The first represents the clinical attitude of valuing active listening and contact between health professionals and patients, who are women with lupus receiving prenatal care, in this study. The second is an existentialist attitude that represents the validation of the problems experienced by each participant. The third attitude is psychoanalytic, manifested as the acceptance and consideration of the unconscious existence, manifest as the psychic expressions between the lines of speech and expressed in the behavior of participants, which requires careful evaluation to be perceived by researchers [3, 4].

### 2.2 Research setting

The research setting was a specialized outpatient clinic, where women with stable diseases attended scheduled appointments for prenatal care [3]. At this specific clinic, approximately 40 pregnant women, who were diagnosed with high blood pressure, sickle cell anemia, cancer, or autoimmune diseases, are treated weekly, and an average of 25 pregnant women with systemic lupus erythematosusare treated annually [3].

This clinic is located in the City of Campinas, associated with the University of Campinas, where medical residents, nursing students, and students studying nutrition and physiotherapy participate in services provided by the Women's Hospital "Professor Doutor José Aristodemo Pinotti" - Center for Integral Attention to Women's Health (CAISM). The Women's Hospital is the second-largest unit at the University of Campinas, with 1200 employees, provides services in over 60 subspecialties, encompassing the complex array of teaching and research activities that exist in the field of health care for women and newborns, and offers diagnostic and therapeutic services, especially in the areas of Gynecology, Obstetrics, Neonatology, and Oncology. The Women's Hospital is maintained using budgetary resources from the University and the public health system in Brazil (SUS). (https://www.unicamp.br/unicamp/saude), and is a reference service center for a region that covers 42 cities (http://www.saude.sp.gov.br/ses/institucional/departamentos-regionais-de-saude/drs-vii-campinas).

The metropolitan region of Campinas is the second-largest metropolitan region in the country, with more than 3.2 million inhabitants [Brazilian Institute of Geography and Statistics (IBGE)], and generated 8.75% of the state's Gross Domestic Product (GDP) in 2016.

The service coverage map can be viewed in Appendix 1.

### 2.3 Environmental adaptation

The researcher's environment is relevant within the context of qualitative research [6,7]. Thus, during the development of our primary study, the first author had been situated in the setting since February 2017, when the observations regarding the functions and relationships within the service were recorded, according to a script, which is available in full in Appendix 2. This script allowed the researcher to undertake and maintain the behavior necessary to develop a rapport with the participants. In addition, the script allowed ample visualization and understanding of the necessary organization and environmental factors during the development of interviews, including the best time to meet with each participant and the best method for maintaining a private environment, protected from interruptions.

### 2.4 Sampling

The sample of participants considered for the primary study was intentionally selected. Our participants had common questions, which covered the pregnancy experience and the diagnosis of lupus, which resulted in their referral and receipt of treatment from the clinic. In addition, we chose to interview the participants during the third trimester of pregnancy, increasing the likelihood that experiences would accumulate during the first two quarters, which appeared to enriched the speeches of the participants, although no studies describing or exploring the perceptions of pregnant SLE patients during earlier periods of pregnancy exist for comparison.

To better highlight the characteristics and particularities of these women, we built a table describing the relevant sociodemographic data, which can be found in “Table 1. Characteristics of the participants” in the article “Perceptions of women with systemic lupus erythematosus undergoing high-risk prenatal care: a qualitative study” [3], available at https://www.sciencedirect.com/science/article/abs/pii/S0266613820300887?dgcid=rss_sd_all5.

### 2.5 Approaching the participants

Data collection was performed through one interview with each participant, between July 2017 to July 2018. The first author established relationships with the interviewees prior to the interview, to create rapport [8], and increase the atmosphere of empathy, trust, and openness for the interview, which can increase the comfort of the participants to respond more freely.

Subsequently, all interviews were conducted by the first author, using a semi-structured script consisting of six open questions, which can be found, in full, in Appendix 3.

The following considerations were applied to the question roadmap:

- The script was created during the research project and received necessary modifications during the environmental adaptation period.
- The objective was to guide the interviewer during the data collection process, but the researcher remained flexible, allowing the free flow of speech with the women being interviewed.
- As a guide, the script was used, as necessary, by the interviewer, who sometimes realized that there was no need to obey the sequence of questions or to ask them all because the interviewee's speech often provided answers without having to pose the questions.

In addition to the interviewees' speeches, we recorded a field diary, containing behavioral and intervening aspects observed during data collection.

A private room was prepared for each interview, containing two chairs facing each other (for the interviewee and interviewer), without tables or utensils that could physically separate the two people or that could indicate any power in this relationship, to provide a relationship without hierarchies and allowing the woman to freely talk about her experience as she saw fit.

The women were invited to participate in the interview on the same day that they had an antenatal medical appointment, to avoid the necessity of additional visits to the clinic.

## 3 Data Analysis

The content analysis was performed in 7 different stages [9], as follows:

1. The notes in the field diary were organized, and the recorded audio of the interviews was literally transcribed by the first author. These notes and transcriptions formed a text that we call the corpus. To present the results, in the text of the primary article, grammatical corrections were made to sections of the chosen discourses, to facilitate the reader's understanding.
2. During this stage, two authors (first author LR and her PhD supervisor FGS) listened to the interviews and read the corpus.
3. The authors registered their impressions on the emerging and significant themes in the right margins of the transcribed text during reading and rereading.
4. During this step, all perceived content (themes and emerging meanings) was organized into categories and subcategories, with the intention of identifying patterns representing significant units and relevant characteristics within the text, considering two important issues: the relevance of the content in each interview, and the frequency [10] with which a given topic appears across all interviews.
5. During the independent analyses performed by two authors, disagreements were identified and discussed with the other authors, until a consensus was reached. In addition to this discussion, this material was presented at meetings for a research group, called Reproductive Health and Healthy Habits (SARHAS), at the University of Campinas, in the same hospital where the care for women with prenatal lupus occurred. Posters and presentations at national and international congresses were also performed, with the intention to discuss and validate this material.
6. The finalization of the screen and the structure of the categories and subcategories, including the construction of a figure that illustrates the thought process of the authors and the discussions between them. This is titled “Figure 1. Comprehending interview reports and the category construction process” and can be found in the article “Perceptions of women with systemic lupus erythematosus undergoing high-risk prenatal care: a qualitative study” [3], available at:
  https://www.sciencedirect.com/science/article/abs/pii/S0266613820300887?dgcid=rss_sd_all
7. All material was validated by peers and members of the SARHAS research group, and NVivo 11 software (QSR International, Melbourne, Australia) was used to organize all material from the beginning of data collection until the end of the study.

In addition, to write the primary article, the consolidated criteria for reporting qualitative research (COREQ) checklist [11] was used, to guarantee the necessary rigor for this type of study.

## 4 Data Validation

Data validation is necessary for qualitative studies to guarantee accuracy [12]. This rigor is understood and must be presented in two ways:

- Internal validation ensures that researchers have the skills to perform the data collection, to establish the necessary relationships with research participants, to understand the eminent meanings in the speeches of the participants, and to analyze the observations made in the field.
- External validation requires that the data, which was analyzed in pairs, be exposed to those with expertise in the theme, through presentations to research groups and scientific events.

For internal validation, we made some necessary reflections and we emphasize the following points:

The first author, who conducted all of the interviews, is a nurse and has experience in hospital care practice and primary care, with a focus on family health, and has accumulated professional experience in relationships with patients and family members in critical situations, such as hospitalizations in a nursing unit or intensive care; situations of social vulnerability, such as hospitalizations in psychiatry and psychosocial care; and situations in which rapid decision-making is required, such as care at a normal birth center.

This experience has allowed her to develop skills in relationships with different hierarchies and to understand how power relationships can be neutralized during different meetings. She (LR) has professional experience as a teacher, accompanying students in theory and care practice, which has helped her to develop the necessary skills to exercise and teach confidentiality and ethics in relationships.

She (LR) has experience with face-to-face interviews, during graduation with nursing academics, facing death and during a master's degree program with parents who experienced the loss of their child in a neonatal intensive care unit, she which made them develop the skills to use semi-structured scripts, dealing with the necessary flexibility to use these instruments and the development of qualitative research on subjects with dense content that may affect the interviewee's feelings and emotions.

However, the interviewee's feelings were welcomed by the research advisor supervisor (F. G. S.) and members of the research group (V. L. P. A. and M. S. S.) during the development of all the work.

For external validation, we have presented our research to the SARHAS group and The 11th international autoimmunity congress, 23th Congress of Gynecology and Obstetrics of Sao Paulo, 2018.

## Ethical Approval

The primary study complies with National Health Council Resolution No. 466 [13] on health research with human beings. It was authorized by the local ethics committee under CAAE no. 68143817.0.0000.5404 [3]. Informed consent of all participants has been obtained in writing.

## CRediT_author_statement

**Larissa Rodrigues:** Conceptualization, Methodology, Data colection, Data analysis, Writing- Original draft preparation, Writing- Reviewing and Editing. **Fernanda Garanhani Surita**: Conceptualization, Methodology, Writing- Original draft preparation, Reviewing and Supervision. **Vera Lucia Pereira Alves:** Data analysis,Visualization, Validation. **Maria Margarida Fialho Sim-Sim:** Data analysis,Visualization, Validation.

## Funding Sources

The first author of this article receives a doctoral scholarship of Improvement of Higher Education Personnel - Brazil (CAPES) no. 88881.188510/2018–01 for the completion of the doctorate that comprises the primary study to which this article is linked.

## Declaration of Competing Interest

All authors declare that there are no known financial interests or interests that may influence or work on this document.

## Appendix Supplementary Materials

Image, application 1
