# Supplementary material for: Qualitative data regarding the experiences of pregnant women with lupus in Brazil
Source: Data Brief. 2020 Dec 2;33:106606. doi: 10.1016/j.dib.2020.106606 (PMC7725719; doi:10.1016/j.dib.2020.106606)
Supplement: Supplementary file 1 [file mmc1.docx]

**Appendix 1**- The service coverage map

Brazil


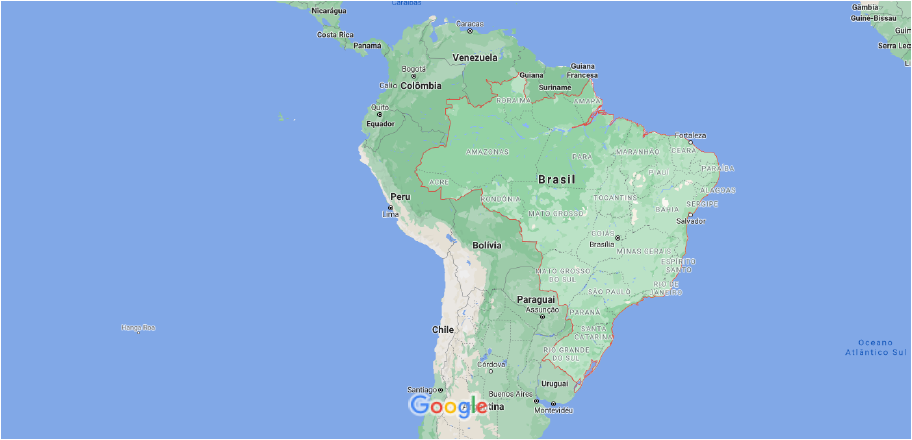


https://www.google.com/maps/place/Brasil/@-13.702797,-69.6865109,4z/data=!3m1!4b1!4m5!3m4!1s0x9c59c7ebcc28cf:0x295a1506f2293e63!8m2!3d-14.235004!4d-51.92528

São Paulo


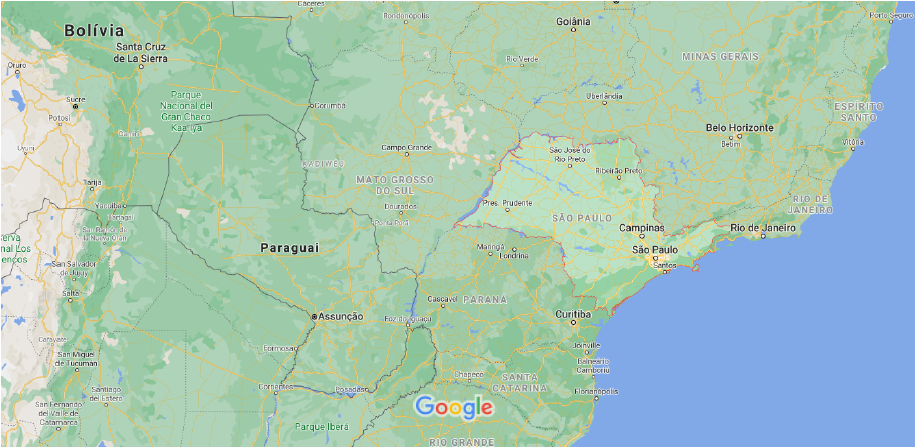


https://www.google.com/maps/place/S%C3%A3o+Paulo/@-22.4836914,-53.1312214,6z/data=!3m1!4b1!4m5!3m4!1s0x94ce597d462f58ad:0x1e5241e2e17b7c17!8m2!3d-23.5431786!4d-46.6291845

Campinas


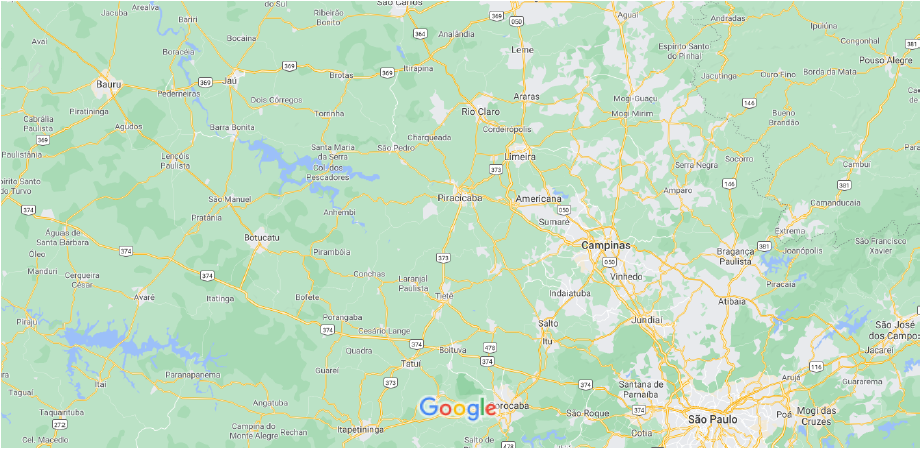


https://www.google.com/maps/place/Regi%C3%A3o+Metropolitana+de+Campinas,+SP/@-22.8276174,-47.6609325,9z/data=!3m1!4b1!4m5!3m4!1s0x94c8cf6de7403fd1:0x6c32eb3480425b62!8m2!3d-22.8131676!4d-47.058302

**Appendix 2-** Script for observing the setting during the environmental adaptation period

Observation script during the researcher's adaptation period at the service

1)Relationships in the waiting room:

2)Medical consultation:

time

relationship

case discussions with supervisors

3) Consultation with nursing:

time

relationships

4) Movement of women within the service:

Examination rooms:

Procedure rooms:

**Appendix 3-** Semi-structured script, with six open-ended questions

1) Tell me how you feel about having an autoimmune disease:

2) Tell me about the experience of being pregnant with this disease:

3) Tell me about your follow-up during pregnancy:

4) Tell me about your relationships since you discovered the disease and pregnancy:

5) Tell me about your life today:

6) Is there something else you want to talk about?
